# Lenvatinib for Anaplastic Thyroid Cancer

**DOI:** 10.3389/fonc.2017.00025

**Published:** 2017-03-01

**Authors:** Makoto Tahara, Naomi Kiyota, Tomoko Yamazaki, Naoko Chayahara, Kenji Nakano, Lina Inagaki, Kazuhisa Toda, Tomohiro Enokida, Hironobu Minami, Yoshinori Imamura, Tatsuya Sasaki, Takuya Suzuki, Katsuki Fujino, Corina E. Dutcus, Shunji Takahashi

**Affiliations:** ^1^Department of Head and Neck Medical Oncology, National Cancer Center Hospital East, Kashiwa, Japan; ^2^Department of Medical Oncology and Hematology, Kobe University Hospital, Kobe, Japan; ^3^Department of Medical Oncology, Cancer Institute Hospital of the Japanese Foundation for Cancer Research, Tokyo, Japan; ^4^Department of Head and Neck Surgery, Cancer Institute Hospital of the Japanese Foundation for Cancer Research, Tokyo, Japan; ^5^Eisai Co. Ltd., Tokyo, Japan; ^6^Eisai Inc., Woodcliff Lake, NJ, USA

**Keywords:** lenvatinib, anaplastic thyroid cancer, tyrosine kinase inhibitor, phase 2, clinical trial

## Abstract

**Background:**

Lenvatinib has been approved by regulatory agencies in Japan, the United States, and the European Union for treatment of radioiodine-refractory differentiated thyroid cancer (RR-DTC). Thyroid cancer, however, is a clinically diverse disease that includes anaplastic thyroid cancer (ATC), the subtype associated with the highest lethality. Effective therapy for ATC is an unmet need.

**Patients and methods:**

This phase 2, single-arm, open-label study in patients with thyroid cancer, including ATC, RR-DTC, and medullary thyroid cancer was conducted from 3 September 2012 to 9 July 2015. Patients received lenvatinib 24 mg daily until disease progression or development of unacceptable toxicity. The primary endpoint was safety, and the secondary endpoint was efficacy, as assessed by progression-free survival (PFS), overall survival (OS), and objective response rate.

**Results:**

At data cutoff, 17 patients with ATC were enrolled. All experienced ≥1 treatment-emergent adverse event (TEAE). The most frequent TEAEs were decreased appetite (82%), hypertension (82%), fatigue (59%), nausea (59%), and proteinuria (59%). Of note, only one patient required lenvatinib withdrawal because of a TEAE, and this TEAE was considered unrelated to lenvatinib. The median PFS was 7.4 months [95% confidence interval (CI): 1.7–12.9], the median OS was 10.6 months (95% CI: 3.8–19.8), and the objective response rate was 24%.

**Conclusion:**

In this study, lenvatinib demonstrated manageable toxicities with dose adjustments and clinical activity in patients with ATC. This clinical activity of lenvatinib warrants further investigation in ATC.

**ClinicalTrials.gov:**

NCT01728623.

## Introduction

Thyroid cancer is a clinically diverse disease that can be classified into differentiated, medullary, and anaplastic thyroid cancer (ATC) subtypes. ATC is an undifferentiated tumor from the follicular epithelium and typically presents with a rapidly enlarging mass as well as dysphagia, neck pain, vocal cord paralysis, and dyspnea (1). Nearly half of all patients with ATC have metastatic disease at initial diagnosis (1). According to the American Joint Committee on Cancer, all patients diagnosed with ATC are classified with stage IV disease (2, 3). Although the incidence is rare among thyroid cancers (1–2%), ATC represents an unmet medical need and is difficult to treat, with an associated mortality rate over 90% (4).

Current therapies for ATC have limited efficacy. Historically, doxorubicin has been considered the most effective treatment, with a response rate of ~20% (5, 6). When combined with other chemotherapies, a higher response rate (50%) can be achieved, but the duration of response is often short (2–5 months) (7, 8). A phase 2 trial of paclitaxel in patients with ATC reported an overall response rate (ORR) of 53%; another trial of carboplatin and paclitaxel in combination with fosbretabulin reported a non-significant increase in overall survival (OS) (9, 10). Combination therapies have also been associated with substantial toxicities, including leukocytopenia, neutropenia, anemia, diarrhea, and vomiting (11).

A total of four multikinase agents are approved for thyroid cancer treatment: cabozantinib (12) and vandetanib (13) for medullary thyroid cancer and sorafenib (14) and lenvatinib for radioiodine-refractory differentiated thyroid cancer (RR-DTC) (15). However, knowledge of their efficacy in ATC is limited. Lenvatinib is a multikinase inhibitor that targets the vascular endothelial growth factor receptor 1–3, fibroblast growth factor receptor 1–4, platelet-derived growth factor receptor-alpha, and RET and KIT proto-oncogenes (16–19). In a preclinical study, lenvatinib demonstrated antitumor activity in mouse ATC xenograft models (19).

Lenvatinib was approved for the treatment of RR-DTC based on results from the phase 3 Study of (E7080) Lenvatinib in Differentiated Cancer of the Thyroid (SELECT) trial (15, 20). In that study, lenvatinib prolonged progression-free survival (PFS) versus placebo [median PFS 18.3 versus 3.6 months; hazard ratio: 0.21, 99% confidence interval (CI): 0.14–0.31; *P* < 0.001] (15). In a separate open-label phase 2 study of lenvatinib in patients with advanced medullary thyroid cancer, the ORR was 36% (95% CI: 24–49%) (21).

This phase 2 study examines the safety and efficacy of lenvatinib in advanced thyroid cancer, including ATC. Although efficacy was not a primary objective, we highlight the efficacy outcome of patients with ATC in this report because encouraging preliminary evidence of antitumor activity was observed in this first trial of lenvatinib treatment for this disease.

## Patients and Methods

### Patients

Eligible patients were aged ≥20 years, had an Eastern Cooperative Oncology Group performance status of ≤2, systolic blood pressure ≤140 mmHg, diastolic blood pressure ≤90 mmHg and had histologically confirmed diagnosis of any of the specified advanced thyroid cancer subtypes (anaplastic, radioiodine-refractory differentiated, or medullary). Patients with ATC must have had evaluable target lesions per the Response Evaluation Criteria in Solid Tumors version 1.1, agreed to hospitalization in cycle 1, and were expected to live for ≥8 weeks after the first dose of study drug. Tumor samples from these patients were subjected to independent pathological review, where possible.

Exclusion criteria included concomitant brain metastases (unless previously treated and clinically stable for ≥1 month prior to screening), bleeding or thrombotic disorders, use of anticoagulants (e.g., warfarin), and proteinuria (urine protein levels of ≥1 g/24 h).

### Study Oversight

This study was sponsored by Eisai Co., Ltd., designed according to the Guidelines for Clinical Evaluation of Anticancer Drugs in Japan and conducted in accordance with the Declaration of Helsinki and local laws. All patients provided written informed consent.

The study and protocol were approved by the Institutional Review Board of each medical institution (National Cancer Center Hospital East, Kashiwa, Japan; The Cancer Institute Hospital of Japanese Foundation for Cancer Research, Tokyo, Japan; and the Kobe University Hospital, Kobe, Japan). The study is registered at http://clinicaltrials.gov (NCT01728623).

Statistical analyses were performed by Eisai Co., Ltd., and Takumi Information Technology Inc., statisticians.

### Study Design

This was a single-arm, open-label, multicenter, phase 2 study to evaluate the safety and efficacy of lenvatinib in patients with advanced thyroid cancer at three study sites in Japan from 3 September 2012 to 9 July 2015. Patients received treatment until progressive disease according to Response Evaluation Criteria in Solid Tumors version 1.1 or development of unacceptable toxicity. Lenvatinib was administered orally at 24 mg once daily on a 28-day continuous cycle. For patients with ATC, tumor assessments were performed at 4, 8, 12, and 16 weeks after the first dose, and every 8 weeks thereafter. Tumor responses were evaluated based on investigator assessment.

Because of historically poor OS, patients with ATC were followed every 4 weeks after treatment discontinuation. Enrollment for this study continued until lenvatinib was approved for unresectable thyroid cancer in Japan, and after approval continued as a post-marketing study until lenvatinib was commercially available at each site. The protocol was amended to include the post-marketing study, but the endpoints and analysis were not changed.

### Safety and Efficacy Analyses

The primary endpoint was the safety of lenvatinib in patients with advanced thyroid cancer, including adverse events (AEs), clinical laboratory results (including urinalysis, hematology, and blood chemistry), vital signs, weight, electrocardiography, and Eastern Cooperative Oncology Group performance status. The assessment of AEs were completed using the Medical Dictionary for Regulatory Activities, and AEs were graded using Common Terminology Criteria for AEs version 4.0.

The secondary endpoints included: PFS (defined as time from the date of first dose to first documentation of disease progression as determined by investigator assessment, or death, whichever occurred first), OS (defined as time from the date of first dose to the date of death from any cause), ORR (defined as the proportion of patients with a complete or partial response), disease control rate (defined as the proportion of patients with complete or partial response, or stable disease, where stable disease must last ≥3 weeks for ATC), and clinical benefit rate [defined as the proportion of patients with complete or partial response, or durable stable disease (stable disease lasting ≥11 weeks for ATC)].

### Statistical Analysis

Statistical analyses were performed using Statistical Analysis System (version 9.2 or later). A sample size of ≥16 was planned, which was estimated to have a probability of >0.8 (or 80%) to detect AEs with a frequency of ≥10%. No adjustments for covariates and no statistical comparisons were planned. Efficacy analyses of PFS and OS were summarized by the Kaplan–Meier method using median time with 95% CI.

## Results

### Patients

**Figure 1:**
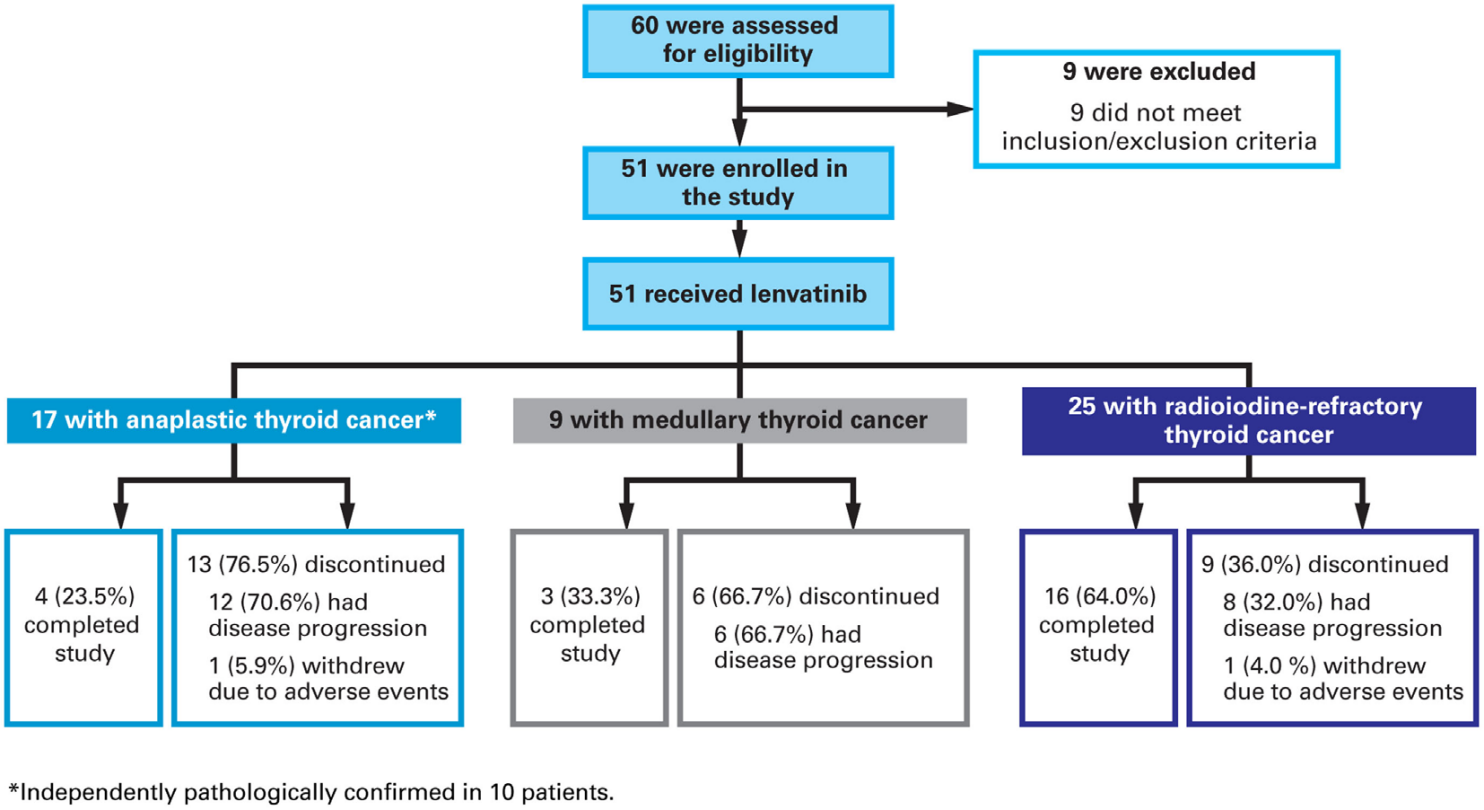
**Patient disposition and reason for discontinuation from study treatment**.

Of the 60 patients screened, nine failed to meet inclusion or met exclusion criteria and 51 were enrolled [Figure 1 (CONSORT diagram)]. There were 17 patients with ATC and results for this patient group are reported here. Tumor samples were available for 10 of these patients and all were retrospectively confirmed to be anaplastic by an independent pathologist. All 17 patients with ATC were included in efficacy and safety analyses. At data cutoff, four (17%) were on treatment, 12 (46%) discontinued because of disease progression, and one (6%) discontinued because of an AE that was deemed unrelated to lenvatinib treatment by the investigator (suicide attempt).

Patient baseline characteristics are summarized in Table 1. All patients were Asian (Japanese) with baseline Eastern Cooperative Oncology Group performance status of 0 or 1, except for two patients with a score of 2. The majority of patients were females (65%) and the median age was 65 years. Most patients (82%) had previous thyroid cancer surgery. Of these patients, seven (41%) had received previous chemotherapy, nine (53%) had received prior external radiotherapy, and two (12%) had not received any prior treatment.

**Table 1:** **Baseline patient characteristics**.

Category	*n* = 17
Median age, years	65.0
Male sex, *n* (%)	6 (35)
Median weight, kg	54.2
Range	39.9–85.3
Eastern Cooperative Oncology Group performance status, *n* (%)	
0	5 (29)
1	10 (59)
2	2 (12)
Stage at diagnosis, *n* (%)	
IVA	4 (24)
IVB	5 (29)
IVC	6 (35)
Unknown	2 (12)
Median baseline lesion diameter, mm	52
Previous therapy, *n* (%)	
Anticancer surgical therapy	14 (82)
Anticancer chemotherapy	7 (41)
Vascular endothelial growth factor therapy	0
Radiotherapy	9 (53)

### Safety

The median duration of lenvatinib treatment was 5.5 months (range: 0.7–33.1). All patients experienced ≥1 treatment-emergent adverse event (TEAE), of which ≥1 was deemed treatment-related by the investigator.

Grade 3 TEAE occurred in 13 (77%) patients and grade 4 TEAE occurred in only one (6%) patient. A summary of common TEAE is shown in Table 2. The most common grade 3 or 4 TEAE were hypertension (*n* = 5; 29%), decreased appetite (*n* = 3; 18%), and thrombocytopenia (*n* = 3; 18%). Serious AEs were reported in 13 patients. There were four fatal serious AEs that occurred within 30 days of the last dose of lenvatinib for this study, three of which occurred in patients with ATC. These three patients died of primary disease progression. All deaths were reported as unrelated to lenvatinib.

**Table 2:** **Treatment-emergent adverse events (AEs) (≥20% of patients in the anaplastic thyroid cancer subgroup)**.

Treatment-emergent AEs, *n* (%)	*n* = 17		
	Any grade	Grade 3	Grade 4
Decreased appetite	14 (82)	3 (18)	0
Hypertension	14 (82)	5 (29)	0
Fatigue	10 (59)	1 (6)	0
Nausea	10 (59)	0 (0)	0
Proteinuria	10 (59)	1 (6)	0
Palmar-plantar erythrodysesthesia syndrome	8 (47)	0	0
Stomatitis	8 (47)	0	0
Constipation	7 (41)	0	0
Decreased weight	7 (41)	0	0
Dysphonia	7 (41)	0	0
Vomiting	6 (35)	0	0
Diarrhea	5 (29)	0	0
Headache	5 (29)	0	0
Increased alanine aminotransferase	5 (29)	0	0
Increased aspartate aminotransferase	5 (29)	0	0
Thrombocytopenia	5 (29)	2 (12)	1 (6)
Peripheral edema	5 (29)	0	0
Arthralgia	4 (24)	0	0
Dehydration	4 (24)	0	0
Epistaxis	4 (24)	0	0
Hypothyroidism	4 (24)	0	0
Malignant neoplasm progression	4 (24)	1 (6)	0
Productive cough	4 (24)	0	0
Pyrexia	4 (24)	0	0

Fifteen (88%) patients required dose reductions and 11 (65%) required dose interruptions because of TEAE. The median time to dose reduction was 0.7 months (range: 0.2–1.4). The most frequent TEAE leading to dose reduction were decreased appetite (*n* = 6; 35%), thrombocytopenia (*n* = 3; 18%), headache (*n* = 3; 18%), fatigue (*n* = 3; 18%), hypertension (*n* = 2; 12%), nausea (*n* = 2; 12%), and palmar-plantar erythrodysesthesia syndrome (*n* = 2; 12%).

### Efficacy

Efficacy outcomes are summarized in Table 3. At data cutoff, eight (47%) patients had received >6 months of lenvatinib treatment. The median PFS was 7.4 months (95% CI: 1.7–12.9) and the median OS was 10.6 months (95% CI: 3.8–19.8). The ORR was 24% and there were four (24%) patients with partial response, 12 (71%) patients with stable disease, and one (6%) patient with progressive disease. Of the four patients with partial responses, four (100%) had previous thyroid cancer surgery, two (50%) had received previous chemotherapy, and two (50%) had prior external radiotherapy. The disease control rate was 94% and clinical benefit was achieved by 12 (71%) patients.

**Table 3:** **Efficacy measures**.

Outcome	*n* = 17
Progression-free survival	
Median [95% confidence interval (CI)], months	7.4 (1.7–12.9)
Overall survival	
Median (95% CI), months	10.6 (3.8–19.8)
Best overall response, *n* (%)	
Complete response	0
Partial response	4 (24)
Stable disease^a^	12 (71)
Progressive disease	1 (6)
Not evaluable	0
Objective response rate, *n* (%)^b^	4 (24)
Disease control rate, *n* (%)^b^	16 (94)
Clinical benefit rate, *n* (%)^c^	12 (71)

*^a^Stable disease is defined as lasting ≥3 weeks*.

*^b^Percentages are based on the patients categorized as evaluable for each parameter. The disease control rate was calculated as complete response plus partial response plus stable disease*.

*^c^The clinical benefit rate was calculated as complete response plus partial response plus durable stable disease (defined as stable disease lasting ≥11 weeks)*.

**Figure 2:**
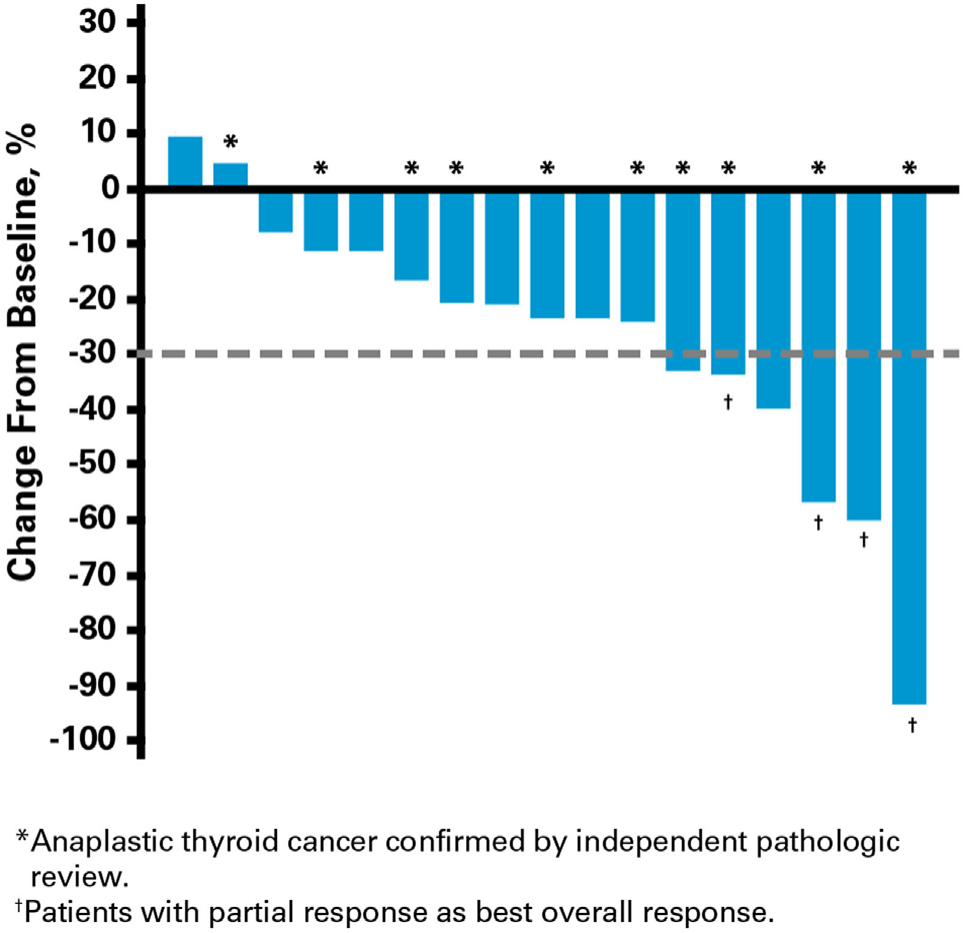
**Percentage change from baseline in summed tumor diameter**.

**Figure 3:**
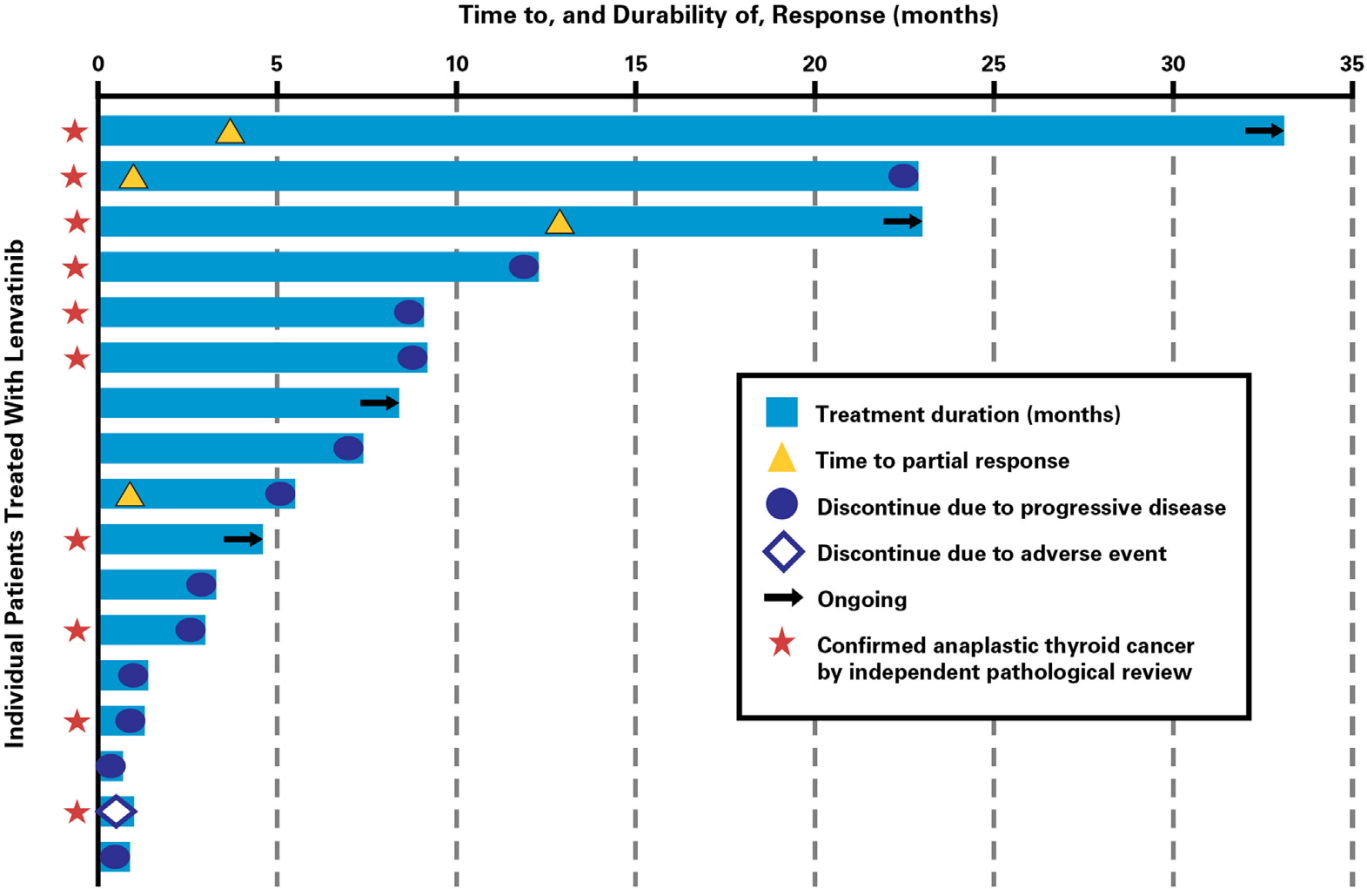
**Time to response and durability of response for patients with anaplastic thyroid cancer treated with lenvatinib**.

**Figure 4:**
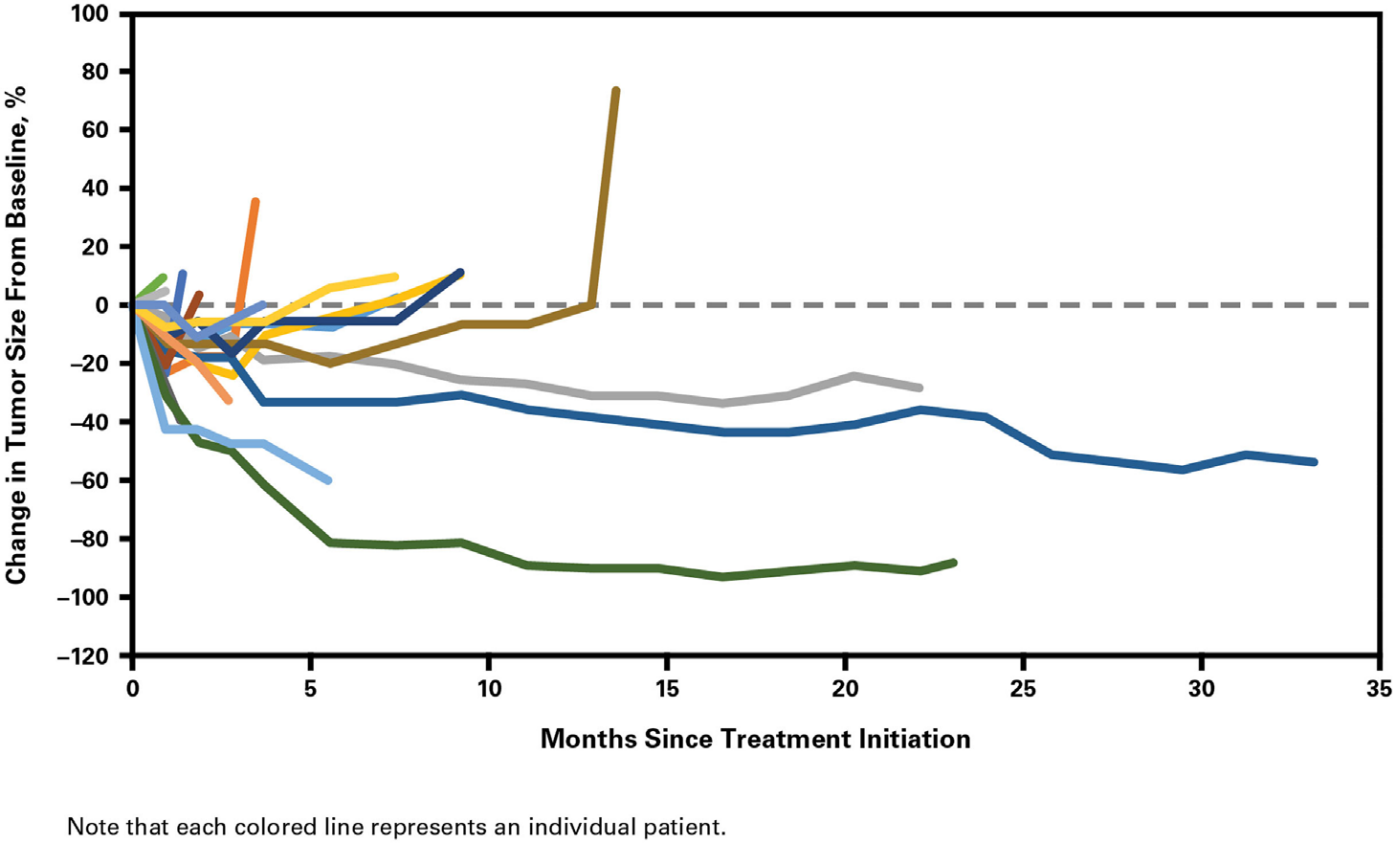
**Percentage change in tumor size from baseline with duration of lenvatinib treatment in patients with anaplastic thyroid cancer**.

**Figure 5:**
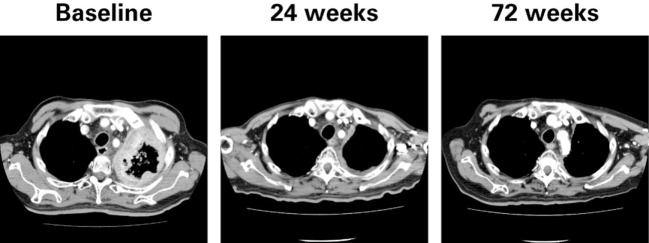
**Computed tomography scans of a representative patient with anaplastic thyroid cancer at baseline and during treatment with lenvatinib**.

Clinical activity was observed in the majority of patients (Figure 2). Patients with ATC demonstrated a durable response to lenvatinib therapy throughout the course of the study (Figure 3). Further, the observed tumor shrinkage in these patients was continuous throughout treatment (Figure 4). These results are illustrated by computed tomography scans of a representative patient with ATC, which revealed remarkable tumor reduction at 24 and 72 weeks post-lenvatinib treatment compared with a baseline scan (Figure 5).

## Discussion

In this phase 2 study, lenvatinib demonstrated a manageable safety profile and meaningful antitumor activity in patients with ATC. The safety profile reported here was comparable to that observed in the phase 2 trial of lenvatinib in medullary thyroid cancer, the phase 3 SELECT trial of lenvatinib in RR-DTC (15, 21), and the Japanese subgroup analysis of the SELECT trial (22). Fatigue, decreased appetite, proteinuria, and hypertension were among the most frequent AEs observed, which are known class effects of vascular endothelial growth factor-targeted treatments (23). Toxicities were effectively managed with dose interruptions and reductions, as demonstrated by having only one patient discontinue treatment because of an AE, which was considered unrelated to lenvatinib.

Responses were observed in all subtypes studied, and most patients experienced tumor shrinkage. We especially note the encouraging responses in patients with ATC, an aggressive form of thyroid cancer with no effective treatment options. This study and the SELECT trial (15) supported regulatory approval of lenvatinib for unresectable thyroid cancer in Japan, and lenvatinib is now commercially available there for the treatment of ATC (24).

As lenvatinib demonstrated striking efficacy in RR-DTC (15), we hypothesized that the ATC subtype may be sensitive to lenvatinib based on evidence that ATC tumors evolved from a background of differentiated thyroid cancer (25). Preclinical studies have also shown that lenvatinib reduced tumor growth in human ATC xenograft mouse models (19). The high vascularity of ATC tumors may also make them more responsive to angiogenesis inhibition by lenvatinib (26).

The ORR in patients with ATC on lenvatinib was noteworthy, with four (24%) patients achieving a partial response, 12 (71%) achieving stable disease, and eight (47%) with durable stable disease. In the context of other investigative or approved agents for the treatment of ATC, which have shown response durations from 2 to 27 months, lenvatinib demonstrated durable response and disease stabilization throughout the course of the study (8, 27). Median OS in patients with ATC who received lenvatinib was 10.6 months, a particularly important finding given that previous studies have reported median OS of <8 months with the use of cytotoxic agents (4, 28, 29). AEs previously reported for paclitaxel included grade 1 stomatitis, alopecia, nausea, diarrhea, and fatigue, grade 2 nausea, gastritis, fever and netropenia, and grades 2−3 neuropathy (9). Doxorubicin is the only approved agent for ATC. Toxicities reported with doxorubicin treatment for advanced thyroid cancer included vomiting and hematologic toxicity (6). Because of the risk of developing cardiotoxicity with increasing total cumulative doxorubicin doses in excess of 400 mg/m^2^, prolonged doxorubicin treatment is problematic (30).

Although there were no bleeding events in the current study, post-marketing surveillance in Japan recently reported some cases of carotid artery and tumor hemorrhage due to rapid tumor shrinkage in patients with ATC treated with lenvatinib. This is further evidence of the antitumor activity of lenvatinib in patients with ATC, although careful administration is especially warranted for patients with tumor invasion of the carotid arteries.

This study has several limitations. First, it is a non-randomized study with a relatively small patient population. Second, there was no biomarker evaluation of tumor samples. Finally, the results for the ATC subgroup represent only one cohort of the study that was not designed to evaluate efficacy of lenvatinib for this subtype as a primary endpoint. In addition, association of biomarkers with efficacy of lenvatinib in ATC was not evaluated in this study, but this will be examined as an exploratory endpoint in ongoing phase 2 trials of lenvatinib in patients with ATC (NCT02657369, NCT02726503). Despite these limitations, the encouraging results observed in this study indicate potentially important improvements in the treatment of advanced thyroid cancer.

In conclusion, lenvatinib therapy demonstrated manageable toxicities, with meaningful and promising antitumor activity in patients with ATC. The results warrant further investigation into the clinical value of lenvatinib for this rare and aggressive malignancy.

## Author Contributions

MT, NK, TY, NC, KN, LI, KT, TE, HM, YI, TSasaki, TSuzuki, KF, CD, and ST read and approved the final manuscript.

## Conflict of Interest Statement

MT had consultant roles at Merck Serono and Eisai, received research funding from Eisai and Boehringer-Ingelheim, and honoraria from Merck Serono and Bristol-Myers Squibb; NK had consultant roles and received research grant support from Eisai, ONO, Boehringer-Ingelheim, and honoraria from Eisai; TY, NC, LI, KT, TE, and YI have nothing to disclose; KN received research grant and honoraria from GlaxoSmithKline and participated in speakers bureau for Novartis, Eisai, MSD, and Serono; HM received grant support and honoraria from Eisai; TSasaki is an employee of Eisai; TSuzuki is an employee of Eisai; KF is an employee of Eisai; CD is an employee of Eisai; ST had consultant roles at Eisai and has received honoraria from Eisai. The reviewer WG and handling editor declared their shared affiliation, and the handling editor states that the process nevertheless met the standards of a fair and objective review.
